# Selective propagation of mouse-passaged scrapie prions with long incubation period from a mixed prion population using GT1-7 cells

**DOI:** 10.1371/journal.pone.0179317

**Published:** 2017-06-21

**Authors:** Kohtaro Miyazawa, Kentaro Masujin, Hiroyuki Okada, Yuko Ushiki-Kaku, Yuichi Matsuura, Takashi Yokoyama

**Affiliations:** 1 Prion Diseases Unit, Division of Transboundary Animal Disease, National Institute of Animal Health, National Agriculture and Food Research Organization (NARO), Tsukuba, Ibaraki, Japan; 2 Nippi Research Institute of Biomatrix, Toride, Ibaraki, Japan; 3 Department of Planning and General Administration, National Institute of Animal Health, NARO, Tsukuba, Ibaraki, Japan; US Geological Survey, UNITED STATES

## Abstract

In our previous study, we demonstrated the propagation of mouse-passaged scrapie isolates with long incubation periods (L-type) derived from natural Japanese sheep scrapie cases in murine hypothalamic GT1-7 cells, along with disease-associated prion protein (PrP^Sc^) accumulation. We here analyzed the susceptibility of GT1-7 cells to scrapie prions by exposure to infected mouse brains at different passages, following interspecies transmission. Wild-type mice challenged with a natural sheep scrapie case (Kanagawa) exhibited heterogeneity of transmitted scrapie prions in early passages, and this mixed population converged upon one with a short incubation period (S-type) following subsequent passages. However, when GT1-7 cells were challenged with these heterologous samples, L-type prions became dominant. This study demonstrated that the susceptibility of GT1-7 cells to L-type prions was at least 10^5^ times higher than that to S-type prions and that L-type prion-specific biological characteristics remained unchanged after serial passages in GT1-7 cells. This suggests that a GT1-7 cell culture model would be more useful for the economical and stable amplification of L-type prions at the laboratory level. Furthermore, this cell culture model might be used to selectively propagate L-type scrapie prions from a mixed prion population.

## Introduction

Scrapie is a transmissible spongiform encephalopathy (TSE) of sheep and goats. TSE, also known as prion disease, is a family of fatal neurodegenerative disorders that affect both humans and animals [1]. The diversity of scrapie prions in sheep has been well investigated [2–6]. Currently, it has believed that sheep scrapie consists of more than 20 strains with different biological phenotypes, including different incubation periods; lesion profiles; biochemical properties of the disease-associated prion protein (PrP^Sc^), a misfolded form of the cellular prion protein (PrP^C^); and neuroanatomical PrP^Sc^ distribution patterns in inbred mice [7–9]. Thus far, there have been no reports of scrapie prions being transmitted to humans. However, a panel of scrapie prions can be transmitted to several lines of transgenic mice overexpressing human PrP^C^ [10]. More recently, scrapie prions were successfully transmitted to primates [11]. Thus, it is important to distinguish and analyze the biological and pathological differences among scrapie prions to determine whether any exhibit zoonotic potential.

We previously reported that two different mouse-passaged scrapie prion types were isolated from a single natural scrapie case (Kanagawa) of sheep by interspecies transmission to mice [4]. These isolates were designated as short-type (S-type) and long-type (L-type) based on their incubation periods and pathologies [4, 5]. Further, we reported that murine hypothalamic GT1-7 cells produced *de novo* PrP^Sc^ in response to L-type prions but not to S-type prions [5]. In this study, we demonstrated through mouse bioassays that the biological properties of L-type prions remained unchanged even after serial passages in GT1-7 cells. Our data suggest that GT1-7 cells can be used to selectively propagate L-type scrapie prions from a mixed prion population during the early transmission of sheep scrapie prions to mouse.

## Materials and methods

### Mouse-passaged sheep scrapie prions in this study

Three mouse-passaged field scrapie prion isolates (Tsukuba-2, Ka/O, and Ka/W) [3, 4] were used in this study. ICR/CD-1 mice infected with L-type prion isolates (Tsukuba-2 and Ka/O) exhibit clinical symptoms of polydipsia and polyuria with a long incubation period (approximately 280 days) and restricted PrP^Sc^ distribution in the brain stem [3, 5]. In contrast, mice infected with the S-type prion isolate (Ka/W) exhibit a short incubation period (approximately 150 days) and marked vacuolation and PrP^Sc^ distribution throughout the entire brain area [4].

### Mouse inoculation

**Fig 1:**
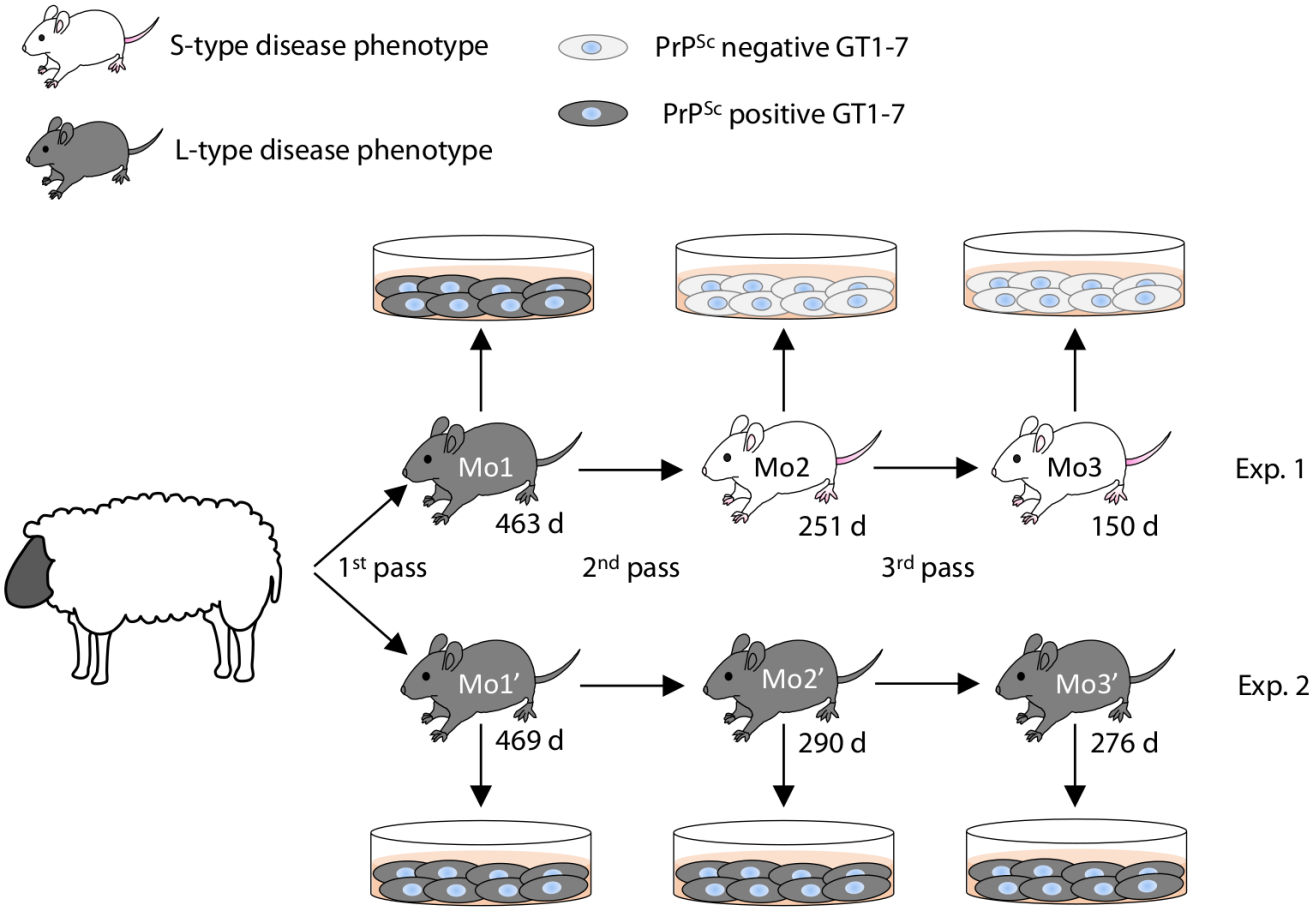
Passage history of scrapie sources used in this study. For the serial passage of scrapie prions in mice, the brain of a single mouse was chosen from the previous passage. The selected mice were named Mo1, Mo2, and Mo3 in Exp. 1 and Mo1′, Mo2′, and Mo3′ in Exp. 2. Mice that clinically and/or pathologically developed L-type disease are shown in grey, while those that developed S-type disease are shown in white. Survival days are indicated underneath each mouse. PrP^Sc^-positive and -negative GT1-7 cells are shown in grey and white, respectively.

Animal experiments were carried out in strict accordance with the regulations outlined in the Guide for the Care and Use of Laboratory Animals of the National Institute of Animal Health (NIAH) and Guidelines for Proper Conduct of Animal Experiments, 2006 by the Science Council of Japan. All animal experiments were performed with the approval of the Animal Ethical Committee and the Animal Care and Use Committee of the NIAH (approval ID: 11–008 and 15–005). For the primary passage, the brain stem of a scrapie affected sheep (Kanagawa) was intracerebrally inoculated into mice (n = 16) as described in our previous report [4]. Two out of the 16 mice were chosen for the secondary passage (referred as Mo1 of Exp. 1 and Mo1′ of Exp. 2 in Fig 1, respectively). Likewise, two mouse brains were selected for the tertiary (Mo2 and Mo2′) and the quaternary (Mo3 and Mo3′) passages. The same brain homogenates were also exposed to GT1-7 cells. All intracerebral inoculations were performed under sevoflurane anesthesia. Three-week-old outbred ICR mice (SLC, Shizuoka, Japan) were inoculated intracerebrally with 20 μL of 10% mouse brain homogenates (2 mg brain equivalent) or GT1-7 cell homogenates (2 × 10^5^ cells). These homogenates were serially diluted before use. Mice were checked daily for the presence of clinical signs, such as emaciation, rough fur, hunched posture, and polyuria. All challenged mice were sacrificed under sevoflurane anesthesia when they presented with clinical disease. Survival period was determined as the time from inoculation to the clinical endpoint or sudden death, at which point brains were collected for analysis. The left hemispheres were immediately stored at −80°C for bioassay and western blot (WB) analysis, and the right hemispheres were fixed with 10% neutral-buffered formalin (pH 7.4) containing 10% methanol for histopathology and immunohistochemistry. Prion phenotypes were assessed in diseased mice according to survival period and/or neuroanatomical PrP^Sc^ distribution patterns [5].

### Exposure of GT1-7 cells to brain homogenates from scrapie-infected mice

Subcultured GT1-7 cells were exposed to brain homogenates of diseased mice as described previously [5]. Briefly, cells were grown on 6-well plates at 10^5^ cells/well. After 2 days of incubation, the medium was replaced with 1 mL medium containing 20 μL of 10% (w/v) brain homogenate as mentioned in the above section. GT1-7 cells exposed to each brain homogenate are referred as GT/Mo1, GT/Mo2, GT/Mo3, GT/Mo1′, GT/Mo2′, and GT/Mo3′. For tissue cell culture endpoint titration assay, cells were exposed to 1 mL medium containing 20 μL of 10-fold serial dilutions of brain homogenate. Another 1 mL of plain medium was added to each well, and cells were incubated for an additional day. Then, the medium was removed, and the cells were washed three times with serum-free medium and seeded into new 6-well plates for the first passage (P1). For subsequent passages, cells were seeded in a new well at a 1:4 dilution every 4 or 5 days, and the remaining cells were cultured in T25 flasks for evaluation of PrP^Sc^ by WB. Each passage and assay was performed twice until the 10^th^ passage (P10).

### Extraction of PrP^Sc^ from brains and cells

Brain homogenates (20%) were mixed with an equal volume of detergent buffer containing 4% (w/v) Zwittergent 3–14 (Merck Japan, Tokyo, Japan), 1% (w/v) Sarkosyl (Sigma-Aldrich Japan, Tokyo, Japan), 100 mM NaCl, and 50 mM Tris-HCl (pH 7.6) and were incubated for 30 min with collagenase (Wako, Osaka, Japan; final concentration of 500 μg/mL) at 37°C. To detect PrP^Sc^ in GT1-7 cells, confluent cells were washed with phosphate-buffered saline (PBS) and then lysed with lysis buffer containing 50 mM Tris-HCl (pH 7.6), 150 mM NaCl, 0.5% (w/v) Triton X-100, 0.5% (w/v) sodium deoxycholate, and 2 mM EDTA. After 2 min of centrifugation at 6,500 ×*g* at 4°C, the supernatant was collected. Then, samples prepared from brains and cells were incubated for 30 min with proteinase K (PK) (Roche Diagnosis Japan, Tokyo, Japan; final concentration of 40 μg/mL) at 37°C. PK digestion was terminated with 2 mM 4-(2-aminoethyl) benzenesulfonyl fluoride hydrochloride (Pefabloc; Roche Diagnostics Japan). Samples were mixed with a 2-butanol/methanol mixture (5:1), and PrP^Sc^ was precipitated by centrifugation at 20,000 *×g* for 10 min at 20°C. Pellets were resuspended in Laemmli sample buffer and subjected to WB.

### Immunoprecipitation

Before immunoprecipitation, concentrations of infected brain homogenates were adjusted to contain approximately the same amount of PK-resistant PrP^Sc^ according to the intensities of the bands obtained by WB. Infected brain homogenates were mixed with brain homogenates prepared from PrP knockout mice to normalize the amount of total protein in each sample. Then, normalized brain homogenates were lysed with an equal volume of buffer containing 0.01% (w/v) Triton X-100, 0.01% (w/v) sodium deoxycholate, 100 mM NaCl, and 10 mM Tris-HCl (pH 7.6) for a final concentration of 5% (w/v). After vortexing, lysed brain homogenates were centrifuged at 500 *×g* for 15 min at 4°C, and the supernatants were collected. Then, supernatants were diluted to a final concentration of 0.5% (w/v) with buffer containing 3% (w/v) Tween 20 and 3% (w/v) Triton X-100 in PBS. Protein G Dynabeads (Life Technologies Japan, Tokyo, Japan) were blocked with 4% (w/v) blocking solution (BlockAce; DS Pharma Biomedical, Osaka, Japan). Diluted supernatants (250 μL) were incubated with PrP^Sc^-specific monoclonal antibody (mAb) 3H6 [12, 13] at a concentration of 1 μg/mL in 500-μL reaction volumes for 2.5 h with rotation at room temperature. Protein G Dynabeads (40 μL) were added and incubated for 2 h on the rotor at room temperature. Beads were washed five times with PBS containing 2% (w/v) Tween 20 and 2% (w/v) Triton X-100. Then, beads were resuspended in Laemmli sample buffer and subjected to WB.

### Western blotting

Samples were electrophoresed on NuPAGE Novex 12% Bis-Tris gels with NuPAGE MOPS-SDS running buffer in accordance with the manufacturer’s instructions (Life Technologies, Carlsbad, CA, USA). Proteins were transferred onto an Immobilon-P membrane (Millipore, Billerica, MA, USA). The blotted membrane was incubated with anti-PrP mAb T2-HRP [14] at 4°C overnight. After washing twice with PBS containing 0.05% (v/v) Tween 20 (PBS-T), signals were developed with a chemiluminescent substrate (SuperSignal; Thermo Fisher Scientific K.K., Yokohama, Japan). For semi-quantitation, blots were imaged using a Fluorchem system (Alpha Innotech, San Leandro, CA, USA) and analyzed using image reader software (AlphaEaseFC; Alpha Innotech) according to the manufacturer’s instructions.

### Histopathology and immunohistochemistry

Formalin-fixed brains were immersed in 98% formic acid to reduce infectivity and then embedded in paraffin wax. Serial sections were stained with hematoxylin and eosin for evaluation of neuropathological changes. After epitope retrieval, PrP^Sc^ immunocytochemistry was performed using the mAbs T1 [14] or SAF84 (Bertin Pharma, Montigny le Bretonneux, France) [15]. Immunoreactions were developed using the anti-mouse universal immunoperoxidase polymer [Nichirei Histofine Simple Stain MAX-PO (M); Nichirei, Tokyo, Japan] as the secondary antibody and were visualized with 3,3′-diaminobenzedine tetrachloride as the chromogen.

## Results

### Susceptibility of GT1-7 cells to scrapie prions during interspecies transmission

As shown in Fig 1, GT1-7 cells produced *de novo* PrP^Sc^ in response to exposure to primary passaged scrapie (Kanagawa)-infected mouse brains in two sets of transmission experiments (Fig 1, Exp. 1 and Exp. 2). However, GT1-7 cells did not produce *de novo* PrP^Sc^ following exposure to Mo2 and Mo3 brains in Exp. 1 (Fig 1, Exp. 1 and S1 Fig). In contrast, *de novo* PrP^Sc^ was detected in GT1-7 cells exposed to Mo2′ and Mo3′ brains in Exp. 2 (Fig 1, Exp. 2 and S1 Fig). To resolve the discrepancy between the two experiments, accumulated prions in the brains and GT1-7 cells were analyzed.

### Heterogeneity of prions in mice infected with sheep Kanagawa scrapie isolate

**Fig 2:**
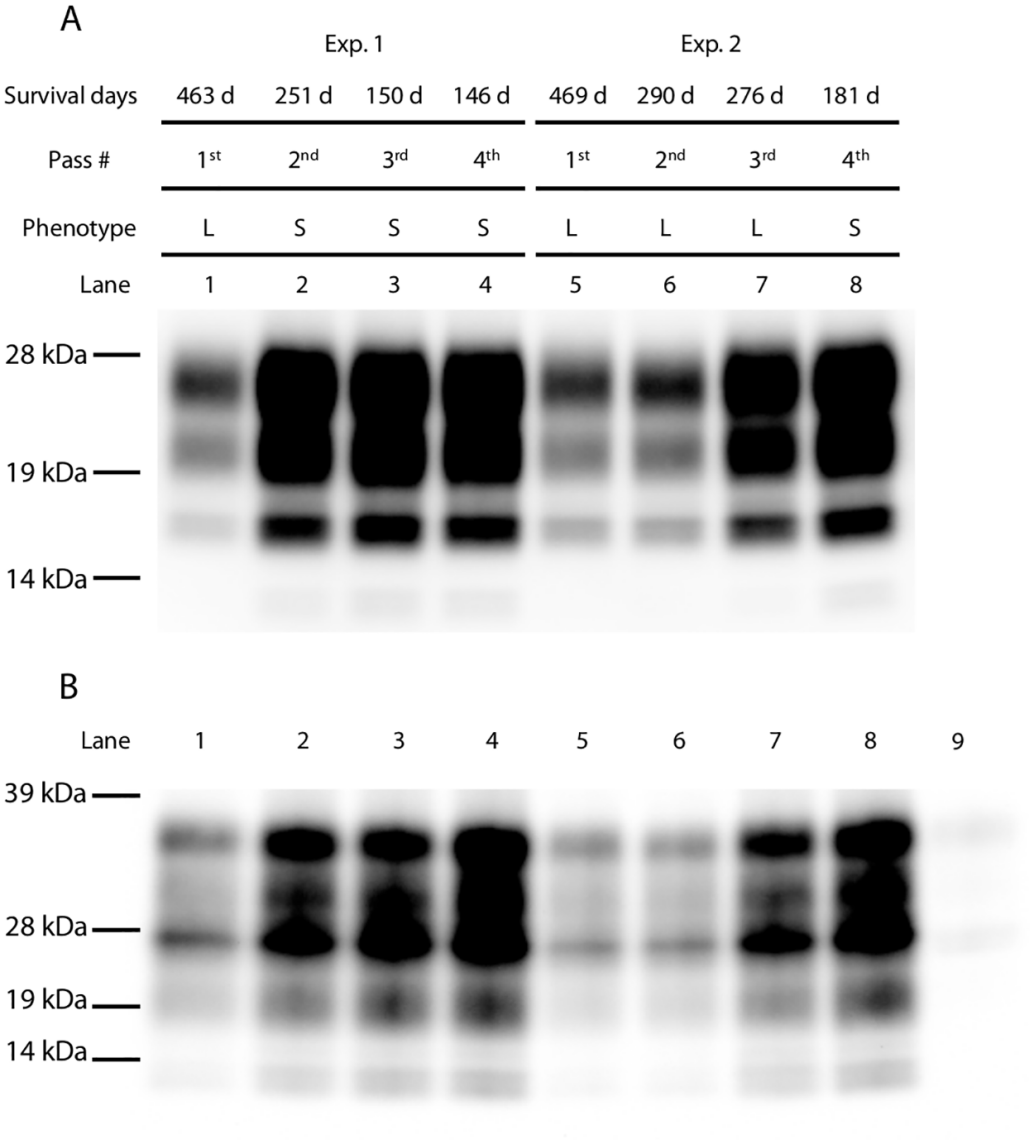
Monitoring PrP^Sc^ accumulation in diseased mice throughout passaging. (A) Representative western blot of PrP^Sc^ in mouse brains at different passages. Experiment number, survival days, passage number, disease phenotype, and lane number are indicated on the top of the panel. Each lane included 0.2 mg brain equivalents. (B) Immunoprecipitation assay with mAb 3H6. Lane number is indicated on the top of the panel. In lane 9, uninfected brain homogenate was probed with mAb 3H6. In all other lanes, PrP^Sc^ was detected with mAb T2-HRP. Molecular markers are shown on the left side of each panel.

As shown in Exp. 2 of Fig 1 and Table 1, mice challenged with the brain homogenates of the sheep scrapie Kanagawa case exhibited L-type disease from the primary to 3^rd^ passages. Because of the species barrier, survival periods in the primary passaged mice were long; therefore, disease phenotype was determined by the neuroanatomical PrP^Sc^ distribution patterns of their brains. In contrast to Exp. 2, the disease phenotype changed from L-type to S-type in Exp. 1 at the 2^nd^ passage. As shown in Fig 2A, mice that developed S-type disease accumulated PK-resistant PrP^Sc^ more rapidly than mice that developed the L-type disease. Weak PrP^Sc^ signals bound to mAb 3H6 were detected in the primary passaged mouse brains in both experiments. The intensity of the PrP^Sc^ signal bound to mAb 3H6 was clearly elevated in the brains of mice that developed S-type disease at the 2^nd^ and subsequent passages (Fig 2B, lanes 2–4). In contrast, PrP^Sc^ signals bound to mAb 3H6 in the brains of mice that developed L-type disease remained weak at the 2^nd^ passage (Fig 2B, lane 6), but the signal intensities gradually increased at the 3^rd^ passage (Fig 2B, lane 7). Lane 8 in Fig 2 shows a mouse whose disease phenotype changed from L-type to S-type at the 4^th^ passage, and its survival period was shortened to 181 days. Both PK-resistant and mAb 3H6-specific PrP^Sc^ signal intensities of this mouse were clearly higher than those detected in the previous passage in Exp. 2. These results may indicate that heterogeneous prions were present in a single mouse challenged with the brain homogenates of scrapie-affected sheep Kanagawa.

**Table 1:** Infection of mice with L- and S-type prions with or without passaging in GT1-7 cells.

	Inoculum ^1^	PrP^Sc^ in inoculum ^2^	Subsequent passages in mice		
			n/n_0_ ^3^	Survival days ^4^	Prion phenotype
Exp. 1 ^6^	Mo1 brain (463 days, L-type) ^5^	+	8/9	196 ± 31.6	S-type
			1/9	289	L-type
	GT1-7 exposed to Mo1 brain	+	9/9	278 ± 5.7	L-type
	Mo2 brain (251 days, S-type)	+	10/10	148 ± 8.9	S-type
	GT1-7 exposed to Mo2	–	8/9	288 ± 36.8	S-type
			1/9	375	L-type
	Mo3 brain (150 days, S-type)	+	5/5	143 ± 4.3	S-type
	GT1-7 exposed to Mo3 brain	–	9/9	246 ±17.6	S-type
Exp. 2	Mo1’ brain (469 days, L-type)	+	5/10	220 ± 7.2	S-type
			5/10	290 ± 6.4	L-type
	GT1-7 exposed to Mo1’ brain	+	9/9	285 ± 15.4	L-type
	Mo2’ brain (295 days, L-type)	+	1/11	195	S-type
			10/11	273 ±13.8	L-type
	GT1-7 exposed to Mo2’ brain	+	9/9	277±14.7	L-type
	Mo3’ brain (276 days, L-type)	+	5/5	191 ± 7.1	S-type
	GT1-7 exposed to Mo3’ brain	+	10/10	280 ± 13.1	L-type

^1^ Inoculum (brain or GT1-7 cells) was intracerebrally injected into ICR mice.

^2^ PrP^Sc^ accumulation was confirmed by western blotting: +, PrP^Sc^ positive;–, PrP^Sc^ negative.

^3^ Number of diseased mice out of total number of challenged mice.

^4^ Survival periods were determined as the time from inoculation to the clinical endpoint or death (mean survival days ± standard deviation).

^5^ Survival periods and prion phenotype of the individual mouse used for the inoculation are indicated.

^6^ Experiment numbers are the same as in Fig 1.

### Disease phenotypes of mice challenged with PrP^Sc^-positive and PrP^Sc^-negative GT1-7 cells

**Fig 3:**
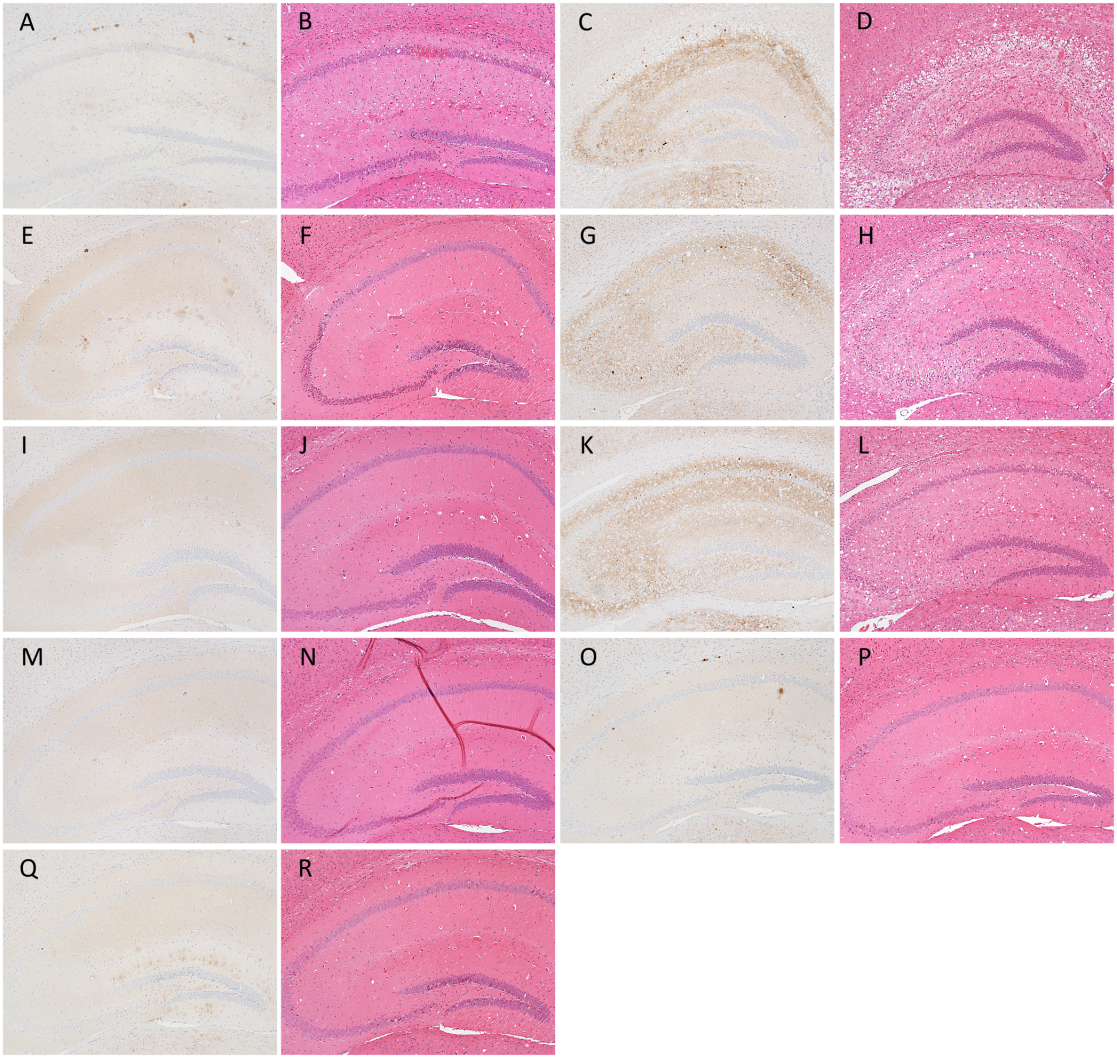
Representative images of PrP^Sc^ deposition types associated with each prion phenotype in ICR mice. Sections of the hippocampus were subjected to immunostaining of PrP^Sc^ and H&E staining. Typical PrP^Sc^ distribution patterns and histopathology of the hippocampus affected with L-type (A and B) and S-type (C and D) prions are shown. Sections of the hippocampi of mice inoculated with cell homogenates prepared from GT/Mo1 (E and F), GT/Mo2 (G, H, I and J), GT/Mo3 (K and L), GT/Mo1 ′ (M and N), GT/Mo2′ (O and P), and GT/Mo3′ (Q and R) were also subjected to immunostaining of PrP^Sc^ and H&E staining. Immunohistochemical detection of PrP^Sc^ was performed using the mAb SAF84.

**Fig 4:**
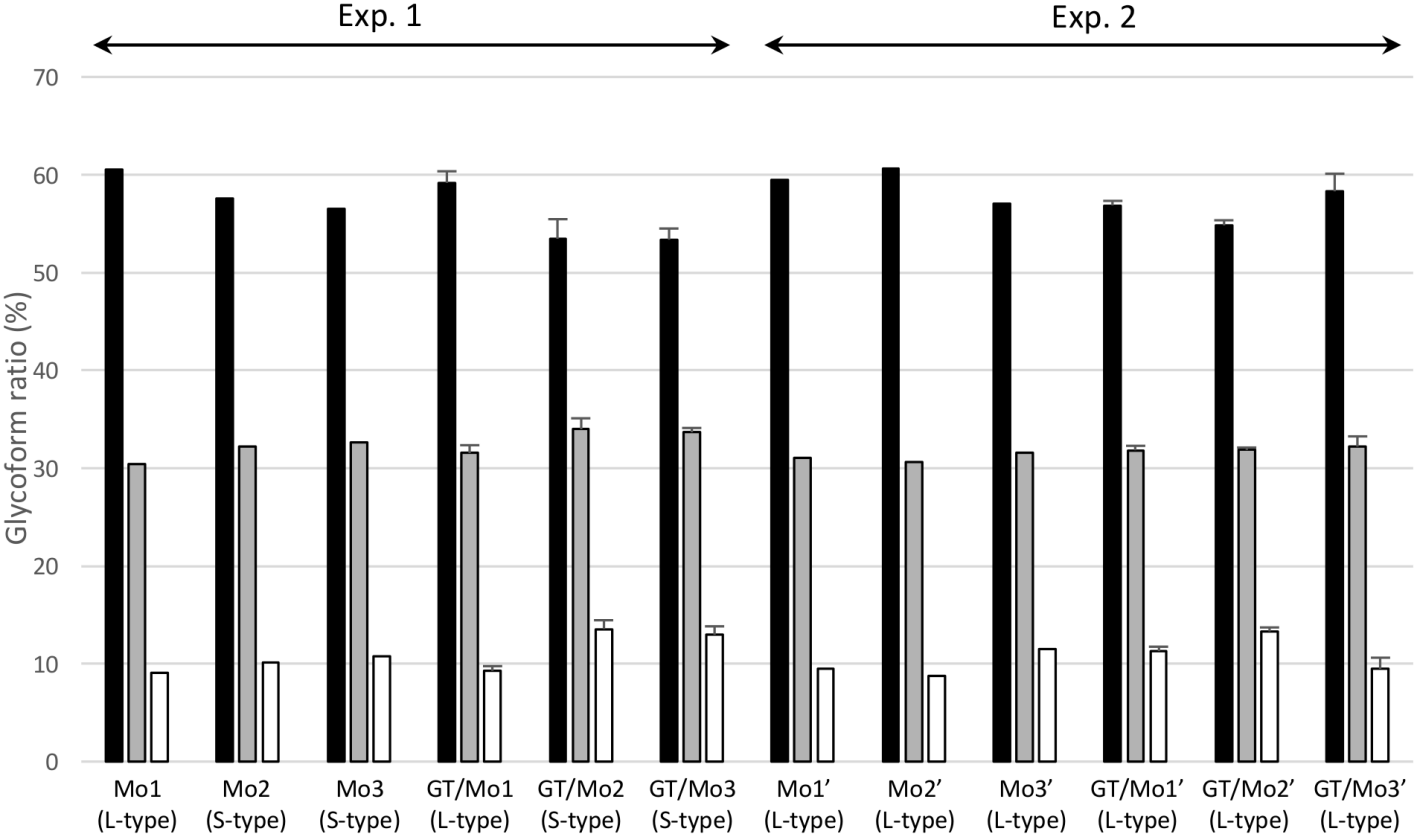
Glycoform profiles of PrP^Sc^ in mice infected with GT1-7 cell culture-derived S-type and L-type prions. PrP^Sc^ glycoform percentages of original mouse brains used for the infection of GT1-7 cells (Mo1, Mo2, Mo3, Mo1′, Mo2′, and Mo3′) were compared to those of mouse brains inoculated with GT1-7 cell homogenates (GT/Mo1, GT/Mo2, GT/Mo3, GT/Mo1′, GT/Mo2′, and GT/Mo3′). Results of GT1-7 cell homogenate-inoculated mice are shown as the mean ± standard deviation (n = 4). The PrP^Sc^ glycoprofile of GT/Mo2-inoculated mice in this graph shows the mean glycoform ratio of the mice that developed S-type disease. The inoculum (brain or GT1-7 cells) was intracerebrally injected into ICR mice. Experiment numbers are the same as in Fig 1.bar graph shows di-glycosylated (black columns), mono-glycosylated (gray columns), and unglycosylated (white columns) forms of PrP^Sc^. L- and S-type at the bottom of each lane indicate the disease phenotypes. PrP^Sc^ was detected with mAb T2-HRP.

To determine whether the biological properties of L- and S-type prions were maintained after serial passages in GT1-7 cell culture, brain homogenates collected from the mice that developed L-type or S-type disease at each passage number were exposed to cells and subcultured until passage #10 (P10), and then these cells were intracerebrally injected into ICR mice. WB analysis demonstrated that GT1-7 cells exposed to the brain homogenates from mice that developed L-type disease harbored PrP^Sc^, while the cells exposed to the brain homogenates from mice that developed S-type disease did not (S1 Fig). All mice challenged with PrP^Sc^-positive GT1-7 cells developed L-type disease with a 100% attack rate (Table 1 and Fig 3) and had similar mean survival periods (277–285 days) when 10% brain homogenates were inoculated (273–290 days) as shown in Table 1. Of the mice challenged with PrP^Sc^-negative GT1-7 cells, only one mouse developed L-type disease (2^nd^ passage of Exp. 1 in Table 1 and Fig 3I and 3J), and the others developed S-type disease with longer incubation periods (2^nd^ and 3^rd^ passages of Exp. 1 in Table 1 and Fig 3G and 3H) as compared to those of mice inoculated with 10% brain homogenates of S-type-diseased mice (Ka/W column of mouse bioassay in Table 2). A clear difference in the glycoprofiles of PrP^Sc^ was not observed between mice challenged with mouse-passaged and cell-passaged prions (Fig 4). These results indicate that the biological features of L-type prions can be sustained in PrP^Sc^-positive GT1-7 cells after serial passages. However, the PrP^Sc^ glycoprofiles of L-type prion-infected GT1-7 cells were clearly different from those of the brains (Fig 4 and S2 Fig), indicating the involvement of not only prion strains (isolates) but also host cellular factors in the PrP^Sc^ glycoform ratio.

**Table 2:** Comparison of the susceptibility of GT1-7 cells and ICR mice to L- and S-type prions.

Assay		GT1-7 at P10 ^1^		ICR mice ^2^	
		PrP^Sc^ accumulation		Survival periods (n/n_0_) ^3^	
Isolate (Phenotype ^4^)		Ka/O (L-type)	Ka/W (S-type)	Tsukuba-2 (L-type)	Ka/W (S-type)
Log_10_ dilution of inoculum ^7^	-1	+ + ^5^	– –	288 ± 5.0 (4/4)	147 ± 3.8 (5/5)
	-2	+ +	N.D ^6^	326 ± 32.8 (5/5)	155 ± 3.1 (5/5)
	-3	+ +	N.D	355 ± 22.1 (3/3)	168 ± 3.4 (5/5)
	-4	+ +	N.D	461 ± 21.5 (4/4)	177 ± 2.9 (5/5)
	-5	+ –	N.D	496 (1/5)	194 ± 4.8 (5/5)
	-6	– –	N.D	>496 (0/5)	246 ± 13.8 (5/5)
	-7	N.D	N.D	>496 (0/5)	203, 234, 235 (3/5)
	-8	N.D	N.D	>496 (0/5)	>496 (0/5)
Infectious units ^8^		10^6.7^ TICD_50_	N.D	10^6.4^ LD_50_	10^8.8^ LD_50_

^1^ PrP^Sc^ accumulation in GT1-7 cells was investigated at 10^th^ passage (P10).

^2^ Mouse endpoint titration assay was conducted.

^3^ Survival periods are shown as mean survival days ± standard deviation. Number of diseased mice out of total number of inoculated mice is shown as n/n_0_.

^4^ Prion phenotypes are indicated.

^5^ PrP^Sc^ accumulation was confirmed by western blotting: +, PrP^Sc^ positive;–, PrP^Sc^ negative. Results of duplicate wells are indicated.

^6^ No data.

^7^ Mouse brain homogenate was diluted from 10^−1^ to 10^−8^. 10−1 indicates 10% brain homogenate.

^8^ Infectious units (TCID_50_ or LD_50_ per gram of brain equivalents) were calculated by Behrens-Karber’s formula.

### Endpoint titration assays of L-type and S-type scrapie prions in GT1-7 cells and ICR mice

To compare the susceptibility to L-type and S-type prions between conventional mice and GT1-7 cells, serial dilutions of L- or S-type prion-infected mouse brain homogenates were used to challenge to ICR mice and GT1-7 cells, respectively (Table 2). Because of the heterogeneity in mouse-passaged L-type scrapie isolates, the Tsukuba-2 mouse-passaged scrapie isolate, which was purified by limiting dilution, was used as a substitute for Ka/O isolates. GT1-7 cells exposed to a 10^−5^ dilution of brain homogenate from a mouse that developed L-type disease produced *de novo* PrP^Sc^ in 1 out of the 2 wells; however, they did not produce *de novo* PrP^Sc^ in response to a 10^−6^ dilution (Table 2 and S2 Fig). Prion titers of the tested brain were estimated at 10^6.7^ TCID_50_/g of brain equivalents (eq). A bioassay using ICR mice challenged with the same dilutions demonstrated that 1 out of the 5 mice clinically and pathologically developed L-type disease in response to the injection of a 10^−5^ dilution, indicating that the tested brain contained 10^6.3^ LD_50_/g of brain eq (Table 2). In contrast, 3 out of the 5 mice challenged with a 10^−7^ dilution of S-type prion-infected brain homogenate developed S-type disease, indicating that this brain contained 10^8.8^ LD_50_/g of brain eq of S-type prions. However, no PrP^Sc^ was detected from GT1-7 cells exposed to the same dilutions or even from those exposed to a 10^−1^ dilution (Table 2).

## Discussion

**Fig 5:**
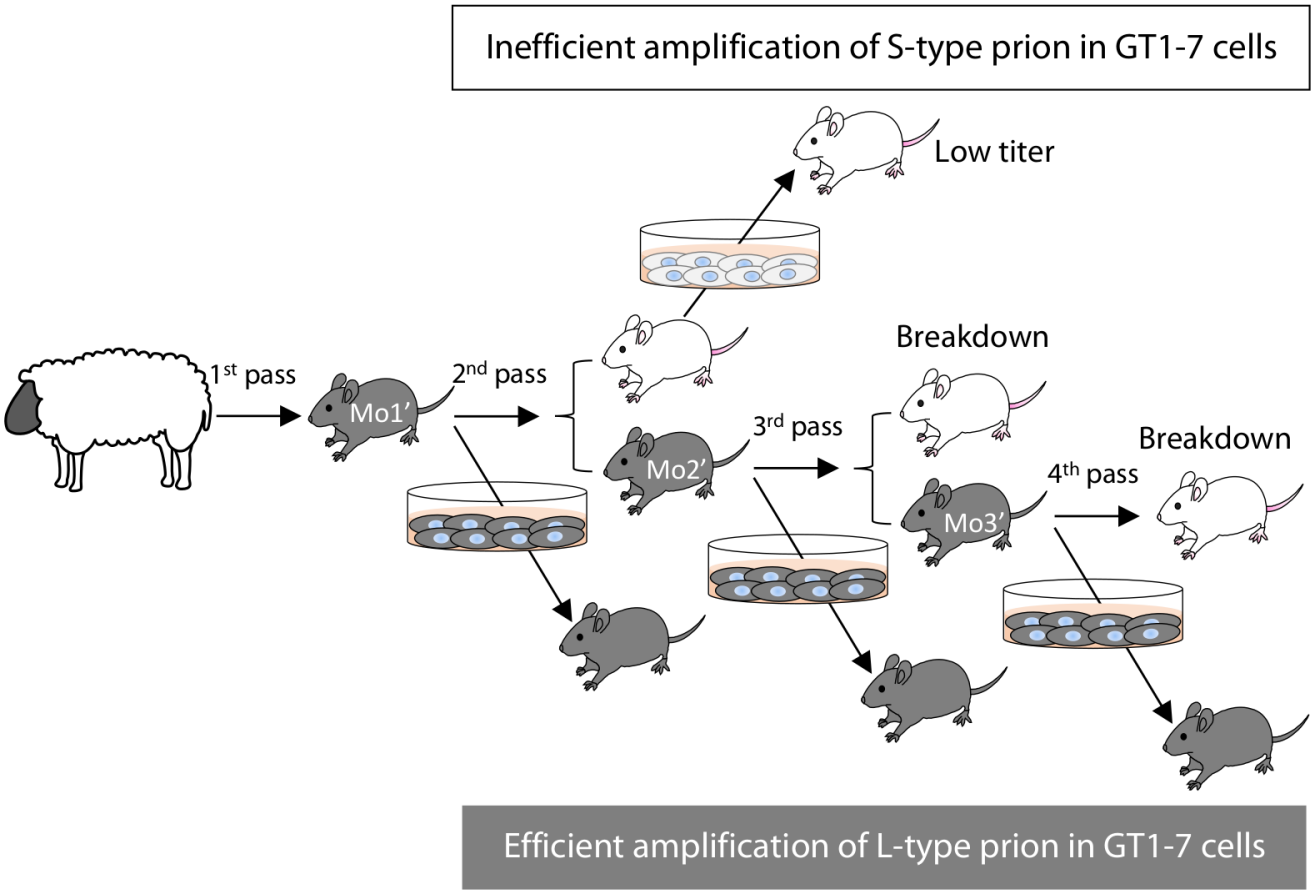
Illustration of breakdown during the transmission of L-type prions in mice and effective amplification in GT1-7 cells. Grey mice and cells indicate the presence of L-type prions, while white mice and cells indicate S-type prions. Mice exhibiting the L-type disease still harbor S-type scrapie prions, resulting in the appearance of mice with the S-type disease at every passage from P2 to P4. GT1-7 cells accumulate PrP^Sc^ in response to L-type prions and can be used to re-propagate mice with L-type prions by injecting mice with cell homogenates. In contrast, GT1-7 cells challenged with S-type prions do not accumulate PrP^Sc^, but they do harbor very low levels of S-type prion infectivity.

At the primary passage, extremely long incubation periods are commonly observed during interspecies transmission. This results from the species barrier. It is well known that incubation periods and neuropathological features are stabilized after a few passages in the same mouse strains [16]. In contrast, it has been reported that the scrapie disease phenotype, including incubation periods, clinical signs, and neuropathology, can suddenly change, even after serial passages in the same mouse strain [17, 18]. This phenomenon is called “breakdown”, and it was observed at the 4^th^ passage of L-type prions in this study (Table 1 and Fig 5). Interestingly, we found that the PrP^Sc^-specific mAb 3H6 preferred to be bound to the PrP^Sc^ that accumulated in mice that developed S-type disease according to immunoprecipitation assays. The amount of PrP^Sc^ bound to mAb 3H6 in a mouse brain selected from the 3^rd^ passage of L-type prions (lane 7 in Fig 2B) tended to be higher than that of the brains selected from previous passages (lanes 5 and 6 in Fig 2B), and the mice inoculated with this brain exhibited breakdown at the 4^th^ passage. These data strongly suggest that the proportions of L- and S-type prions in the brain are altered during serial transmission in mice. In addition, mouse endpoint titration assays for both S- and L-type prions enable us to speculate that S-type prions replicated faster than L-type prions in mice, as mice that received 10^2.8^ infectious units of S-type prions developed the disease earlier than those that received 10^6.4^ infectious units of L-type prions (Table 2). This indicates the possibility that S-type prions easily become dominant throughout subsequent passages in mice when both prions co-exist in a single mouse brain. Experimental mice are routinely used to amplify scrapie prions. We propose that close attention must be paid in order to maintain L-type prions in laboratory mice. In contrast to mice, GT1-7 cells efficiently amplify PrP^Sc^ and sustain high prion infectivity in response to L-type prions but not to S-type prions. Moreover, the biological properties of L-type prions remain unchanged after serial passages in GT1-7 cells. Taken together, this cell culture model could be convenient for the stable amplification of L-type prions.

It requires over 280 days to obtain the bioassay results regarding L-type prions even when mice are challenged with 10% brain homogenates. However, using PrP^Sc^ accumulation as an index, GT1-7 cell culture assay could produce results in approximately 60 to 70 days. In addition, the sensitivity of tissue cell culture assay to L-type prions was comparable to that of mouse bioassay. In contrast, the amplification of S-type prions in GT1-7 cells was extremely inefficient (Table 2). An unexpected finding of this study was that the mice challenged with PrP^Sc^-negative GT1-7 cells developed S-type disease (2^nd^ and 3^rd^ passages in Exp. 1 of Table 1). These cells were subcultured more than 10 times before injection into mice. It is likely that the original brain homogenate exposed to GT1-7 cells was diluted to a level below the detection limit of the mouse bioassay; that is, we cannot exclude the possibility that very low levels of S-type prions continue to replicate even in PrP^Sc^-positive GT1-7 cells. Further study will be needed to resolve this issue. Nevertheless, we here demonstrated that the susceptibility of GT1-7 cells to L-type prions was at least 10^5^ times higher than that to S-type prions. We conclude that GT1-7 cells would be useful for the selective propagation of L-type prions. Recently, the relationship between the seeding activity of PrP^Sc^ and prion infectivity has been revealed through the use of protein misfolding cyclic amplification (PMCA) and real-time quaking-induced conversion (RT-QuIC) [19–22]. Therefore, combining the tissue cell culture assays developed here with PMCA and/or RT-QuIC may provide new opportunities for the purification of L-type prions from a mixed prion population.

It remains unclear why L-type prions are efficiently amplified in GT1-7 cells, unlike in the mouse brain. One possibility is that conformational differences between L- and S-type prions directly affect their effective replication in GT1-7 cells. Indeed, our immunoprecipitation assays may indicate that L-type prions have structurally different PrP^Sc^ conformations from S-type prions. The other possibility is that host-encoded cofactors, which may be lacking in GT1-7 cells, are required for the efficient replication of S-type prions. Previous reports done by others also support the selective susceptibility of neuronal populations in animals and in cell culture models to specific prion strains or isolates [23–27]. Our data may suggest the possibility that prion strain-specific cofactors are differentially expressed among various cells. Cofactors for prion replication, such as phospholipids and polyanions, have been mainly identified in *in vitro* PrP^Sc^ amplification studies [28–30]. Further study is required to clarify the cofactors that are clearly involved in the effective replication of specific prions in *in vivo* models.

## Supporting information

S1 Fig Susceptibility of GT1-7 cells to diseased brains taken from different mouse passages. Representative western blot of GT1-7 cells exposed to diseased brains derived from different mouse passages (1^st^ to 3^rd^). Experiment number, passage number, protein loaded (μg), and lane number are indicated at the top of each lane. GT1-7 cells were collected at P10, and PrP^Sc^ was detected with mAb T2-HRP. Mr indicates the protein marker. Molecular markers are shown on the left. Three laboratory scrapie prion strains (22L, ME7, and Chandler) were used as positive and negative infection controls. As previously reported [5], GT1-7 cells were susceptible to 22L and Chandler but not to ME7.
(TIF)

S2 Fig PrP^Sc^ glycoprofiles of GT1-7 cells infected with L-type prion. Glycoform ratios of GT1-7 cells exposed to Mo3′ brain homogenates were calculated at passage #8 (P8) and #10 (P10). PrP^Sc^ was detected with mAb T2-HRP. The bar graph shows di-glycosylated (black columns), mono-glycosylated (gray columns), and unglycosylated (white columns) forms of PrP^Sc^. Values are expressed as the mean ± standard deviation (n = 3).
(TIF)

S3 Fig Tissue cell culture endpoint titration assay of GT1-7 cells exposed to brain homogenates of passage 3 mice exhibiting the L-type disease. Representative western blot of GT1-7 cells exposed to serial dilutions of brain homogenates from mice with the L-type disease phenotype at P10. Isolate name and prion phenotype of the inoculum are indicated at the top. The log_10_ dilution factor of the brain homogenate and the amount of protein loaded (μg) are also indicated at the top of each lane. PrP^Sc^ was detected with mAb T2-HRP. Molecular markers are shown on the left.
(TIF)
