# Supplementary figures and images for: Selective propagation of mouse-passaged scrapie prions with long incubation period from a mixed prion population using GT1-7 cells
Source: PLoS One. 2017 Jun 21;12(6):e0179317. doi: 10.1371/journal.pone.0179317 (PMC5479544; doi:10.1371/journal.pone.0179317)

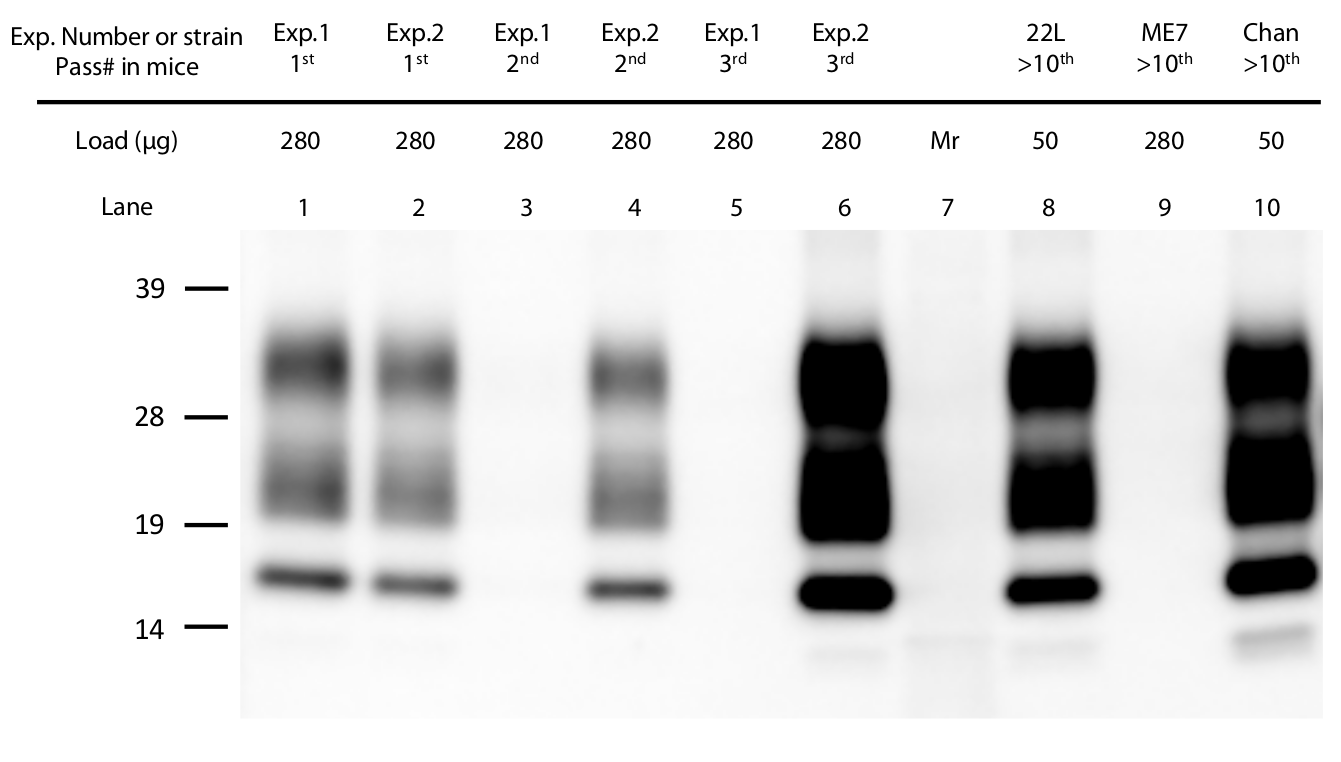

Supplement: S1 Fig — Representative western blot of GT1-7 cells exposed to diseased brains derived from different mouse passages (1st to 3rd). Experiment number, passage number, protein loaded (μg), and lane number are indicated at the top of each lane. GT1-7 cells were collected at P10, and PrPSc was detected with mAb T2-HRP. Mr indicates the protein marker. Molecular markers are shown on the left. Three laboratory scrapie prion strains (22L, ME7, and Chandler) were used as positive and negative infection controls. As previously reported [5], GT1-7 cells were susceptible to 22L and Chandler but not to ME7. (TIF) [file pone.0179317.s001.tif]

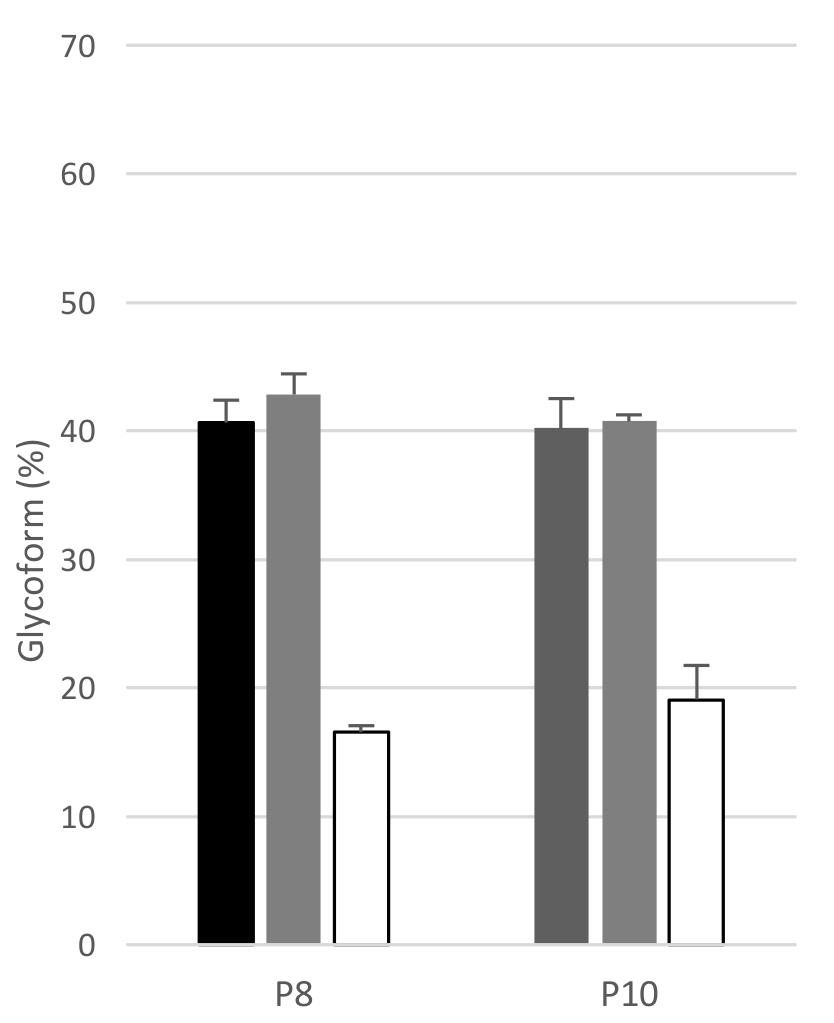

Supplement: S2 Fig — Glycoform ratios of GT1-7 cells exposed to Mo3′ brain homogenates were calculated at passage #8 (P8) and #10 (P10). PrPSc was detected with mAb T2-HRP. The bar graph shows di-glycosylated (black columns), mono-glycosylated (gray columns), and unglycosylated (white columns) forms of PrPSc. Values are expressed as the mean ± standard deviation (n = 3). (TIF) [file pone.0179317.s002.tif]

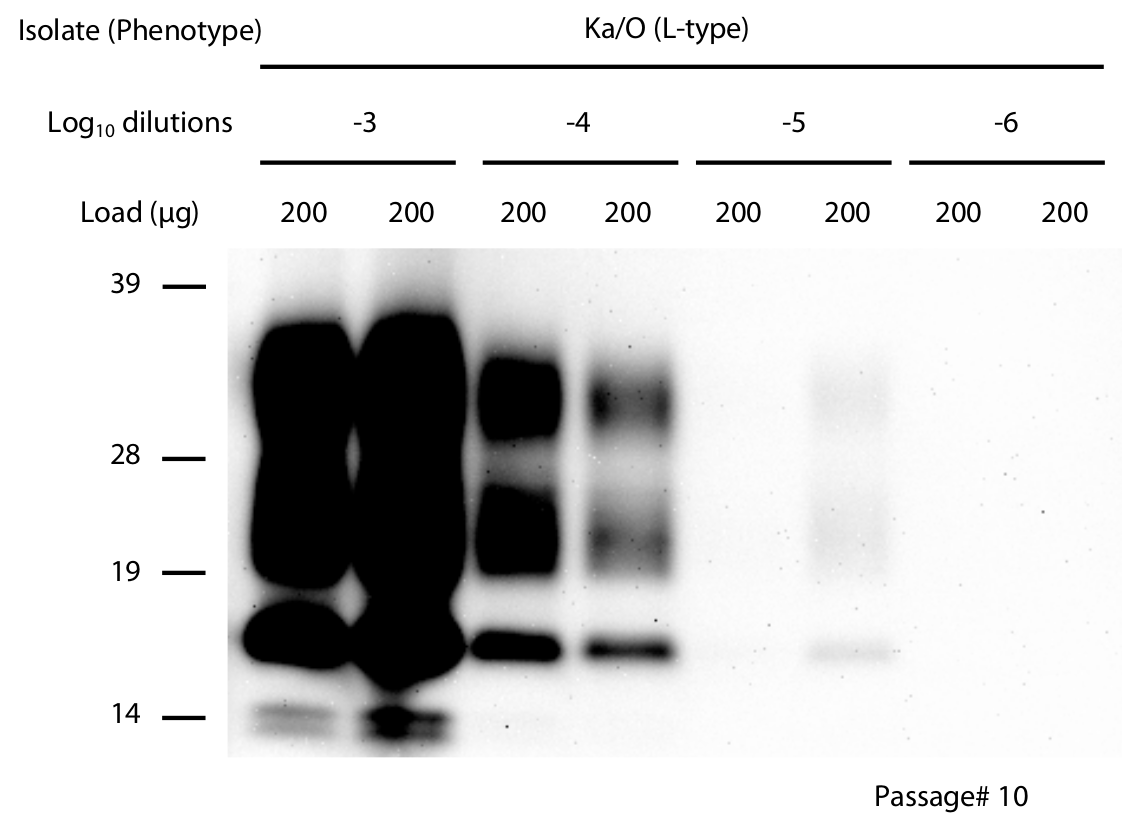

Supplement: S3 Fig — Representative western blot of GT1-7 cells exposed to serial dilutions of brain homogenates from mice with the L-type disease phenotype at P10. Isolate name and prion phenotype of the inoculum are indicated at the top. The log10 dilution factor of the brain homogenate and the amount of protein loaded (μg) are also indicated at the top of each lane. PrPSc was detected with mAb T2-HRP. Molecular markers are shown on the left. (TIF) [file pone.0179317.s003.tif]
